# Controlling the Quantum State with a time varying potential

**DOI:** 10.1038/s41598-017-13313-3

**Published:** 2017-10-16

**Authors:** Sebastián Carrasco, José Rogan, Juan Alejandro Valdivia

**Affiliations:** 1 Departamento de Física, Facultad de Ciencias, Universidad de Chile, Casilla 653, Santiago, 7800024 Chile; 2 Centro para la Nanociencia y la Nanotecnolgía, CEDENNA, Santiago, 9170124 Chile

## Abstract

The problem of controlling the quantum state of a system is investigated using a time varying potential. As a concrete example we study the problem of a particle in a box with a periodically oscillating infinite square-well potential, from which we obtain results that can be applied to systems with periodically oscillating boundary conditions. We derive an analytic expression for the frequencies of resonance between states, and against standard intuition, we show how to use this behavior to control the quantum state of the system at will. In particular, we offer as an example the transition from the ground state to the first excited state of the square well potential. At first sight, it may be counter intuitive that we can control the state of such a quantum Hamiltonian, as the Schrödinger equation conserves the norm of the wave function. In this manuscript, we show how that can be achieved.

## Introduction

Since the development of Quantum Theory, the study of time dependent quantum systems has been of wide interest^1–4^, and in particular, the control the quantum phenomena has been an implicit task. As Dong and Petersen wrote, “*One of the main goals in quantum control theory is to establish a firm theoretical footing and develop a series of systematic methods for the active manipulation and control of quantum systems*”. Such capability could be used to store information in the system, or in an array of them, using many states to store information as the experimental limits allow us^5^.

With this in mind, in this letter we revisit the problem of a particle in a one-dimensional infinite square-well potential, but with a moving wall which we use to drive the system systematically from one quantum state to another. Furthermore, we will derive analytic expressions that characterize this process. Our scheme may be characterized as a bi-linear quantum control strategy^6^ that can be used to develop similar strategies in the context of other systems where the dynamics follows a Schrodinger equation; not necessary in the quantum regime, such as quantum optics or in Bose-Einstein condensates; but also in water waves, optical fibers, planar waveguides, among others.

To do so, we start by showing that by oscillating the wall at certain frequencies we can produce an oscillation between two predefined states, in a manner that is similar to the results obtained by Lenz *et al*.^7^ in a different system. It is important to mention that Glasser *et al*.^8^ also reported a similar behavior for the infinite square-well potential but they didn’t found the rule to do so since they focused their study on low, intermediate, and high frequencies of the wall motion; finding adiabatic, chaotic, and periodic behavior respectively. We will see, as a by-product of our quest, that the classical macroscopic effects of the Fermi acceleration process^9^ and chaotic dynamics for large amplitude oscillating walls^10^ could also be associated with the respective dynamics in the quantum domain. Our problem of interest is a quantum variant of the famous Fermi acceleration problem^11^, but with impenetrable walls instead of inhomogeneous magnetic fields. In this sense, the idea behind this equivalent Fermi acceleration process can be illustrated by making a ping pong ball bounce between a table and a paddle moving towards the table.

The first work about our problem at hand, as far as we can tell, was made by Doescher and Rice^12^, where they solved the Schrödiner equation for the case of a moving wall at constant magnitude of velocity motion. After that article, the problem has been discussed in several publications using different approaches, such as the adiabatic approximation, sudden expansion or contraction of the wall, rapidly varying square well width, etc.^1–4^.

Following our quest, we now demonstrate that is it possible to construct a sort of effective stroboscopic Hamiltonian for this time-dependent periodic problem, as it is done for the classical particle with the oscillating wall^9,10^. This operator emerges naturally when we study the time evolution operator that can give us the oscillatory response of the system, so that in essence we are constructing a simile of Bloch’s Theorem^13^, but for the time domain, in the case of oscillating boundary conditions. This stroboscopic Hamiltonian determines the interaction of the accessible states and the long term evolution of the system. It is worth mentioning that recently a work by Di Martino *et al*.^14^ suggested that a unitary transformation to the fixed wall problem can be made. Before, Pereshogin and Pronin^15^ showed that this moving wall problem can be cast to an effective Hamiltonian and a Berry phase. Even earlier, Munier *et al*.^16^ constructed a generalized canonical transformation, which suggested why this effective Hamiltonian could be constructed.

## Method

### How to use the wall to connect quantum states of a particle in a square-well potential

To simplify the analysis, we will first study the time evolution of a particle in an infinite square well potential with an oscillating wall moving with a constant magnitude of velocity. Later on, we will show that the results also apply to more general oscillations. For the case of a particle of mass *m* between two rigid walls at positions *x* = 0 and *x* = *L*_0_, we can choose a Hilbert space with the basis {|*φ* ^*n*^〉} given by the solution of the time-dependent Schrödinger equation, namely

1 $$\langle x|{\phi }^{n}(t)\rangle =\sqrt{\frac{2}{{L}_{0}}}{e}^{-i\frac{{n}^{2}{\pi }^{2}\hslash }{2m{L}_{0}^{2}}t}\,\sin \,(\frac{n\pi x}{{L}_{0}}),$$

where *n* is the enumeration index of the base vector, and *ħ* is Planck’s constant. We can follow the time evolution of every admissible state by simply projecting that state into this basis at for example *t* = 0.

Similarly, Doescher and Rice^12^ found a solution for the problem where the right wall moves with constant magnitude of velocity *v*. In that case, we can choose as the complete time-dependent Hilbert space basis the set of eigenstates $\{|{\varphi }_{v,L(t)}^{n}\}$ given by

2 $$\langle x|{\varphi }_{v,L(t)}^{n}(t)\rangle =\sqrt{\frac{2}{L(t)}}{e}^{i\frac{{n}^{2}{\pi }^{2}\hslash }{2mvL(t)}}{e}^{\frac{ivm{x}^{2}}{2\hslash L(t)}}\,\sin \,(\frac{n\pi x}{L(t)}).$$

As before, we can follow the dynamics of every admissible state by simply projecting that state into this basis at for example *t* = 0. It becomes important to note that the time evolution of this eigenstate is simply parameterized by the change of *L*(*t*) = *L*_0_ + *vt*, so we can forget the time and think the evolution in terms of the box length. At the same time, we note that for *v* < 0, the kinetic energy of the particle in a given eigenstate goes as *E*_*n*_ ~ *L*(*t*)^−2^ plus a correction which is function of *v*, so that the energy increases as the wall shrink the system as *L* = *L*_0_ − *vt*, in a similar way a ping pong paddle accelerates the bouncing ball in a Fermi acceleration process.

Let us consider a wall oscillation from *L*_0_ to *L*_0_ + *d* and back at a constant magnitude of velocity defining an effective frequency Ω. In that case, we must consider three different basis, {|*φ* ^*n*^〉} and $\{|{\varphi }_{\pm v,L(t)}^{n}\rangle \}$, where *v*_±_ = ±*ωd*/*π* are the speeds of the right wall when it moves right and left, respectively. This is a simple example that illustrates a more general result, as we will explain later in this letter. Note that all these basis are orthogonal, consequently, the change of basis is unitary.

With this in mind, we can study the time evolution of every state. Initially, the quantum state of the system should be a linear combination of {|*φ* ^*n*^〉}, but when the wall moves right the system will evolve according to the basis $\{|{\varphi }_{+v,L(t)}^{n}\rangle \}$, so that we project the state in this basis to give account of that. In a similar way, when the box size becomes *L*_0_ + *d* the system will evolve according to the basis $\{|{\varphi }_{-v,L(t)}^{n}\rangle \}$ until the box size becomes *L*_0_ again, and then we can go back to the basis {|*φ* ^*n*^〉}. After these three projections, we can encounter the system in a different state. The dynamics evolves the system in the same way for every oscillation because the eigenstates are parameterized only by *L*(*t*).

We can give account of this process by a discrete time evolution operator, that evolves the system over one oscillation, namely, ${\check{S} }|\psi (t=\mathrm{0)}\rangle =|\psi (t=2\pi /\omega )\rangle$. The matrix elements of this operators can be expressed on the {|*ϕ* ^*n*^〉} basis as follows

3 $$\langle {\varphi }^{n}|{\check{S} }|{\varphi }^{p}\rangle =\sum _{k=1}^{\infty }\,\sum _{l=1}^{\infty }\,\langle {\varphi }^{n}|{\varphi }_{-v,{L}_{0}}^{l}\rangle \langle {\varphi }_{-v,{L}_{0}+d}^{l}|{\varphi }_{+v,{L}_{0}+d}^{k}\rangle \langle {\varphi }_{+v,{L}_{0}}^{k}|{\varphi }^{p}\rangle .$$

Note that ${\check{S} }$ is unitary because the product of two or more unitary operators is unitary. Furthermore, this discrete stroboscopic time evolution operator can have its own set of eigenstates and eigenvalues, where the eigenvalues are complex numbers of modulus equal to one, hence for *N* oscillations may be written as ${\lambda }_{n}^{N}={e}^{i2\pi N{\alpha }_{n}/\hslash \omega }$. Inspired by the form of the eigenvalues, it may be useful to think of this stroboscopic operator as coming from a Hamiltonian ${\mathop{H}\limits^{\check{}}}_{s}$, that we call the stroboscopic Hamiltonian, with eigenvalues *α*_*n*_. Note that ${\mathop{H}\limits^{\check{}}}_{s}$ is an Hermitian operator. It’s worth to mention that this decomposition presented here of the evolution operator is similar to the one presented in the work of Novo *et al*.^17^.

Hence, the time evolution can be described by the effective Hamiltonian ${\mathop{H}\limits^{\check{}}}_{s}$, and it’s expected that $[{\mathop{H}\limits^{\check{}}}_{s},{\mathop{H}\limits^{\check{}}}_{0}]\ne 0$, where ${\mathop{H}\limits^{\check{}}}_{0}$ is the Hamiltonian of the static problem. In that case, we will have oscillations on the value $\langle {\mathop{H}\limits^{\check{}}}_{0}\rangle$, as a result of Ehrenfest theorem^18^, so that we can define Bohr frequencies that determine the long time evolution of the system, and the mixing of the states. Below, we will analyze the conditions that produce $[{\mathop{H}\limits^{\check{}}}_{s},{\mathop{H}\limits^{\check{}}}_{0}]\ne 0$. Furthermore, we must recall from classical dynamics that the Hamiltonian is not necessarily equal to the energy so that the eigenvalues of ${\mathop{H}\limits^{\check{}}}_{s}$ don’t need to correspond to the energy of the state.

It’s important to point out that we can follow the same argument for any periodic oscillation because every oscillation of the wall can be understood as many steps with *d* → 0.

So far this treatment is exact but difficult to track analytically so that in order to make explicit calculations let us consider a particular case where $d/{L}_{0}\ll 1$ and the oscillations are made with the constant magnitude of velocity strategy discussed above. In this case, we can solve (3) and later compute the matrix elements in the basis of the static problem, namely,

4 $${S}_{k,n}=\langle {\phi }^{k}|{\check{S} }|{\phi }^{n}\rangle ={\delta }_{kn}{e}^{-2i\frac{{k}^{2}\pi }{\bar{\omega }}}+i{C}_{k,n}{e}^{-i\frac{({k}^{2}+{n}^{2})\pi }{\bar{\omega }}}\,(1-{e}^{-i\frac{({k}^{2}-{n}^{2})\pi }{\bar{\omega }}})+{\mathscr{O}}(\bar{d}{}^{2})\,$$

where $$\bar{\omega }=2m\omega {L}_{0}^{2}/{\pi }^{2}\hslash $$, $$\bar{d}=d/{L}_{0}$$, and

5 $${C}_{k,n}=\frac{\bar{\omega }\bar{d}}{{\pi }^{2}}\frac{4{(-1)}^{k+n}kn\pi }{{({k}^{2}-{n}^{2})}^{2}}.$$

It’s worth noting that at least at first order of $\bar{d}$, all the phenomenology is given by this two dimensionless parameters, $\bar{d}$ and $\bar{\omega }$. Another point is that, as we established before, the operator is unitary as can be seen by computing ${{\check{S} }}^{\dagger }{\check{S} }=1$ to first order.

The probability transference between states as defined by the eigenstates of the evolution operator are slightly different from the static ones. That must happen if the non-diagonal elements are not zero. Indeed, the squared modulus of the non-diagonal elements are proportional to

6 $${|{S}_{k,n}|}^{2}\propto \frac{1}{2}-\frac{1}{2}\,\cos \,(\frac{({k}^{2}-{n}^{2})\pi }{\bar{\omega }}).$$

For a given value of ${\bar{\omega }}_{k,n}^{\ell }$, this quantity has a maximum for a certain value of *k* and *n*, so the state *k* and *n* becomes connected. Note that this condition is symmetric under change of *k* and *n*, as ${\bar{\omega }}_{k,n}^{\ell }$ can be rewritten as

7 $${\bar{\omega }}_{k,n}^{\ell }=\frac{{k}^{2}-{n}^{2}}{2\ell -1}\,{\rm{for}}\,{\rm{some}}\,\ell \in {\mathbb{N}},$$

which is the resonance condition.

It becomes also important to note that the dynamics is determined by the non-diagonal elements, and hence the phenomenology should become noticeable after many oscillations because the non-diagonal elements are proportional to $\bar{d}$. Therefore, it makes sense to think in terms of a Stroboscopic Hamiltonian for small $\bar{d}$ values. We will analyze below what happens for larger $\bar{d}$ values.

Before we end this section of the manuscript, we must point out that our discrete time evolution operator could be obtained via a Floquet approach^19^, which is completely equivalent to the one used here. In fact, the Floquet exponents *λ*_*n*_ are related to the eigenvalues of the stroboscopic Hamiltonian, namely, *λ*_*n*_ = *iα*_*n*_/*ħ*. However, in the case of a wall moving with a constant magnitude velocity, we have an expression for the intermediate states, so that constructing the evolution operator in terms of the inner products of these states is straight forward.

### How to use the connection between states to control the state of the system at will

We now turn to our most fundamental task, that is to drive the system from one quantum state to another, and keep it there. At first sight, it might be quite counter intuitive that it is possible to control the state of the system represented by the stroboscopic Hamiltonian, as the Schrödinger equation conserves the norm of the states. The key for our quest is to understand how the system manages to remember when a probability is increasing and when is decreasing on a resonance process. The information is stored in the phase of the amplitude of every eigenstate, information that vanishes when we take the squared moduli. As the Schrödinger equation doesn’t have inertia, it is the only place where it can be stored. Therefore, if we can control the phase of every state, we can control which probability is increasing and which is not. To do so we can let the system evolve certain amount of time with no oscillation until we have the correct relative phase between the states we want to affect, and then restart the oscillation of the wall to drive the system to any asymptotic state we want to obtain.

## Results

### Connecting states

**Figure 1:**
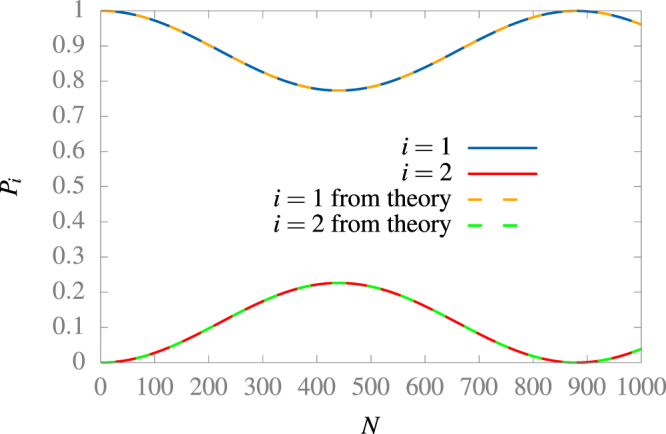
Probability of finding the system on the states |*φ* ^1^〉 and |*φ* ^2^〉 after *N* oscillations at frequency $\bar{\omega }=3$, at constant magnitude of velocity motion. To do this calculation we computed numerically the evolution operator on the static box basis for the first 2 states and use $\bar{d}=0.001$. The sum of the state amplitudes is always bigger than 0.9997, the rest of energy goes to larger states.

Let us use our analysis to connect the state |*φ* ^1^〉 with the state |*φ* ^2^〉. As we determined in Eq. (6) the wider maximum is at $\bar{\omega }={\bar{\omega }}_{\mathrm{1,2}}^{1}=3$, so, this frequency should provides the easiest way to tune this resonance. We compute the dynamics applying *N* times the evolution operator calculated numerically at all orders of $\bar{d}$, and then compute the probability of finding the system at the states |*φ* ^1^〉 and |*φ* ^2^〉, as we observe in Fig. 1, where we use |*φ* ^1^〉 at *t* = 0 as the initial state. In this Figure, we find oscillations between the two states of the static box. Note that the time scale of the phenomena is almost three orders of magnitude slower than the oscillation of the box, in accordance with the size of $\bar{d}$.

We can explain the curve if we suppose that only the block of the operator ${\mathop{H}\limits^{\check{}}}_{s}$ that involves the first and second state is non-diagonal on the basis of eigenstates of ${\mathop{H}\limits^{\check{}}}_{0}$, namely $|{\lambda }_{1}={e}^{i{\beta }_{1}}\rangle =\,\cos \,\theta |{\phi }_{1}\rangle +{e}^{i\varphi }\,\sin \,\theta |{\phi }_{2}\rangle$, $|{\lambda }_{2}={e}^{i{\beta }_{2}}\rangle =-{e}^{-i\varphi }\,\sin \,\theta \,|{\phi }_{1}\rangle +\,\cos \,\theta \,|{\phi }_{2}\rangle$, and $|{\lambda }_{j}={e}^{i{\beta }_{j}}\rangle =|{\phi }_{j}\rangle$ for *j* > 2. If we follow the dynamics of the state |*φ*_1_〉 through *N* oscillations, the probability of finding the system on the eigenstate |*φ*_1_〉 and |*φ*_2_〉 are

8 $${P}_{1}(N)={\cos }^{4}\,\theta +{\sin }^{4}\,\theta +2\,{\cos }^{2}\,\theta \,{\sin }^{2}\,\theta \,\cos \,[({\beta }_{1}-{\beta }_{2})N]$$

and *P*_2_(*N*) = 1 − *P*_1_(*N*), respectively. Now, if we compute numerically the matrix elements of ${\mathop{H}\limits^{\check{}}}_{s}$ on the basis of eigenstates of ${\mathop{H}\limits^{\check{}}}_{0}$, we can solve for *θ* and make a prediction of the curve, that we see as the dashed lines of Fig. 1. Hence, we find that our reduced model works quite accurately, which suggest that we can split the matrix in blocks for our analysis as long as $\bar{d}$ is small, as we did. In the same way, a similar analysis can be made for three or more eigenstates, in the case of larger $\bar{d}$ values.

**Figure 2:**
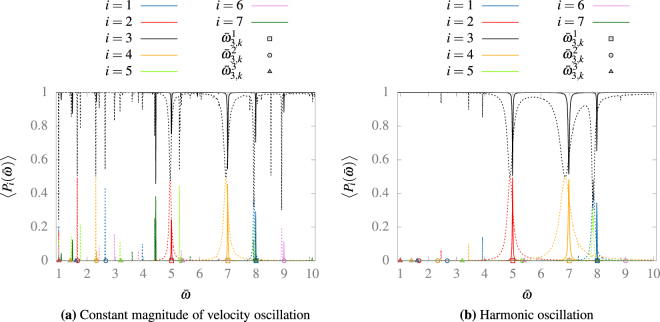
Average probability of finding the system on the states |*φ* ^*i*^〉 after 100,000 oscillations at different frequencies $\bar{\omega }$, in a harmonic motion, with $\bar{d}=0.001$, in continuous line, and $\bar{d}=0.01$, in dashed line. The expected resonance frequencies given by Eq. (7) are shown with points. In the left we computed numerically the evolution operator on the static box basis for the first 15 states in the cases that the wall moves at constant magnitude of velocity motion. In the right we computed the time evolution of the first 7 states for wall that moves harmonically. The sum of all probabilities is always bigger than 0.99 in the left and 0.999 in the right.

In order to test Eq. (7) we study the average probability of the first 7 states, starting from the state |*φ* ^1^〉 for different values of $\bar{\omega }$ and $\bar{d}$, as we show in Fig. 2a. We observe that the position of all the maxima fit well with our predictions, at least for $\ell =1,\,2,\,3$; and $\bar{d}=0.001$. It is interesting to note that the maxima move a little for $\bar{d}=0.01$, but this can be expected since Eq. (7) is only valid at first order. It is worth noticing that different resonances have different widths but all widths increase with the value of $\bar{d}$. Furthermore, when we use bigger values of $\bar{d}$, we can observed oscillations (interactions) between three or more states, which may be related to the chaotic behavior that appears in the classical regime^10^, remembering that chaotic dynamics is expected to appear quite naturally when be observe three wave resonances^20^. We will analyze this in a future manuscript. Finally, we observe that not all peaks have the same maximum average probability.

We claim that our analysis is valid for different types of oscillations, no just magnitude constantly moving walls. Indeed, we can numerically solve the Schrödinger equation for a particle in a periodically varying box, but with a more continuously varying oscillation. To do so, we use a similar algorithm to the one proposed by Doescher *et al*.^12^ Let us introduce the *ansatz*

9 $$\psi (x,\,t)=\sum _{n=1}^{\infty }\,{A}_{n}(t)\sqrt{\frac{2}{L(t)}}\,\sin \,(\frac{n\pi x}{L(t)}),$$

in the time-dependent Schrödinger equation. We find that

10 $$\frac{{\hslash }^{2}{k}^{2}{\pi }^{2}}{2m{L}^{2}}{A}_{k}=i\hslash \frac{\partial {A}_{k}}{\partial t}-i\hslash \frac{\dot{L}(t)}{2L(t)}{A}_{k}-i\hslash \,\sum _{n=1}^{\infty }\,\frac{2n\dot{L}(t){A}_{n}}{\pi L(t)}\,{\int }_{0}^{\pi }\,\sin \,(kx)\,\cos \,(nx)\,x\,dx,$$

where *L*(*t*) is the varying position of the right wall. If we truncate the sum to *m* terms, we can solve this numerically. We can construct a figure equivalent to Fig. 2a, but for a harmonic oscillation of the form,

11 $$L(t)={L}_{0}+d\,[1-\,\cos \,(\omega t)],$$

as we see in Fig. 2b. In this case, we find the same resonances between states at the same frequencies predicted by our approach with the constantly moving wall. Hence, in this Figure we corroborate that our analysis, as described by Eq. (7) holds for such continuous type of oscillations, at least with one frequency. It’s important to recall that this explains in an analytic manner the results of Glasser *et al*.^8^. Oscillations with multiple frequencies will be studied in detail in a future publication.

For large amplitudes, we expect the system to become more classical, as states with larger energy become involved, and eventually reach the chaotic regime of the Fermi-Ulam problem^9^. This transition is of course not trivial, as the Floquet approach becomes more involved. We plan to study this problem in detail in a future manuscript.

### Controlling the quantum state

**Figure 3:**
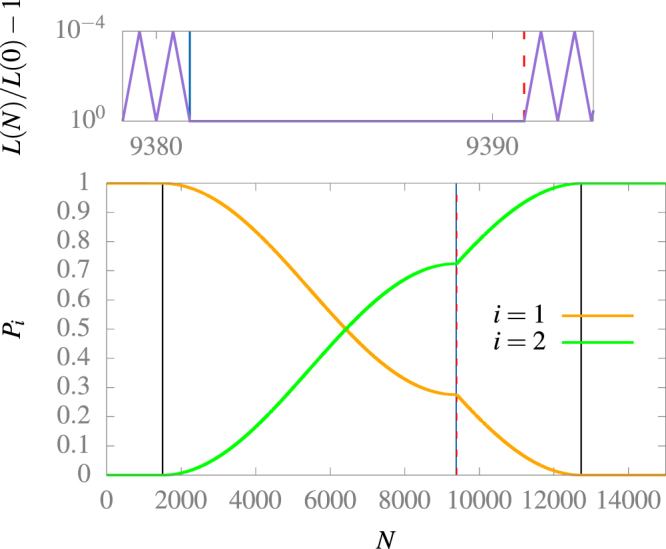
Probability of finding the system on the states |*φ* ^1^〉 and |*φ* ^2^〉 after *N* oscillations at frequency $\bar{\omega }=2.9998$ and $\bar{d}=0.0001$, and box size near to the instant where the box stops. To do this calculation we computed numerically the evolution operator on the static box basis for the first 15 states. The sum of all probabilities is always bigger than 0.99999998. At the end *P*_1_ = 0.00001 and *P*_2_ = 0.99918.

In Fig. 3 we use that procedure to obtain the state |*φ* ^2^〉, asymptotically, starting from the state |*φ* ^1^〉. To do so, we start with no wall oscillation and at the first vertical black line we start oscillating the wall using a constant magnitude of velocity motion with $\bar{\omega }=2.9998$, as predicted by our previous analysis. At the blue vertical line the probability of finding the system in the state |*φ* ^1^〉 has a minimum. At that time, we stop the wall oscillation and wait, for a time equivalent to 9.94 oscillations of the wall, until we acquire the necessary relative phase. At that point, shown as the vertical red dashed line, we start oscillating the wall again until we get to another minimum, shown as the last vertical black line. Here, we stop the wall oscillation and find the system in a state that has the probability *P*_2_ = 0.99918 of being in the state |*φ* ^2^〉. This process can be optimized to increase the probability *P*_2_ if necessary.

## Conclusions and Discussion

In summary, we have studied the phenomenology of a particle in a squared-well potential with an oscillating wall. We found a previously reported oscillation between states for this system and we constructed an analytic description by considering small and constant magnitude of velocity oscillations. Additionally, we found the frequencies that appear in this phenomenon using a simple two level model, at least for $\bar{d}\ll 1$. We then go even further and showed that our results are even more general since they apply to more continuous oscillations.

Using our previous results, we developed a method to use this phenomenon to control systematically the quantum state of the system by controlling the motion of the oscillating wall. This procedure may be used to store information in the system, or in an array of them, using many states to store information as the experimental limits allow us.

At first sight, it may be very surprising that this time-dependent problem can be studied in terms of a time-independent problem using the stroboscopic Hamiltonian. Similarly, it is quite counter intuitive that we can control the state of the system using only this Hamiltonian, because an important property of the Schrödinger equation is that the time evolution doesn’t change the norm of the states. But in this case, the eigenstates are a combination of “non-moving wall” states so that an appropriate combination with two Hamiltonian can make this happen.

Finally, we plan to study in a future work the chaotic transition that we expect to happen at bigger values of $\bar{d}$, where several frequencies resonate at the same time, and that may be related to the transition to the chaotic dynamics that appears in the classical regime, which has been studied in previous works. Although the general setting of this manuscript is about the fundamentals of quantum mechanics, we can imagine possible applications in quantum optics or Bose-Einstein condensates, where it could be possible to submit these type of systems to a control strategy by varying the boundary conditions; for example, in the later case, by tuning the laser trapping system^21^. Of course in such systems, we would expect to see the appearance of nonlinear terms, that could also have applicability in systems like water waves, optical fibers, planar waveguides, among others^22,23^.
